# Thermographic evaluation of acupoints in lower limb region of individuals with osteoarthritis: A cross-sectional case-control study protocol

**DOI:** 10.1371/journal.pone.0284381

**Published:** 2023-04-14

**Authors:** Bao-Hong Mi, Xue-Zhou Wang, Jing-Wen Yang, Guang-Xia Shi, Wen-Zheng Zhang, Li-Na Jin, Li-Sha Yang, Dong-Hua Liu, Si-Bo Kang, Hang Zhou, Yi-Ran Wang, Li-Qiong Wang, Jian-Feng Tu

**Affiliations:** 1 International Acupuncture and Moxibustion Innovation Institute, School of Acupuncture-Moxibustion and Tuina, Beijing University of Chinese Medicine, Beijing, China; 2 Dongzhimen Hospital, Beijing University of Chinese Medicine, Beijing, China; 3 Jiaodong Community Health Service Station, Beijing, China; 4 Xiaoguan East Street Community Health Service Station, Beijing, China; 5 Deluyuan Community Health Service Station, Beijing, China; King Khalid University, SAUDI ARABIA

## Abstract

**Purpose:**

Acupuncture has been widely used in the treatment of knee osteoarthritis (KOA), but the selection of acupoints is indeterminate and lacks biological basis. The skin temperature of acupoints can reflect the state of local tissue and may be a potential factor for guiding acupoint selection. This study aims to compare the skin temperature of acupoints between KOA patients and the healthy population.

**Study design and methods:**

This is a protocol for a cross-sectional case-control study with 170 KOA patients and 170 age- and gender-matched healthy individuals. Diagnosed patients aged 45 to 70 will be recruited in the KOA group. Participants in the healthy group will be matched with the KOA group based on mean age and gender distribution. Skin temperature of 11 acupoints (ST35, EX-LE5, GB33, GB34, EX-LE2, ST34, ST36, GB39, BL40, SP9, SP10) will be extracted from infrared thermography (IRT) images of the lower limbs. Other measurements will include demographic data (gender, age, ethnicity, education, height, weight, BMI) and disease-related data (numerical rating scale, pain sites, duration of pain, pain descriptors, pain activities).

**Discussion:**

The results of this study will provide biological evidence for acupoint selection. This study is a precondition for follow-up studies, in which the value of optimized acupoint selection will be verified.

**Trial registration:**

ChiCTR2200058867.

## Introduction

Knee osteoarthritis (KOA) is one of the most common musculoskeletal diseases and an important cause of chronic pain and lower limb disability among elderly individuals [1]. As one of the most popular nonpharmacologic therapies, acupuncture has long been used in the treatment of KOA in many countries. The Osteoarthritis Research Society International (OARSI) guidelines [2] and the American College of Rheumatology (ACR) guidelines [3] recommend acupuncture conditionally, while the National Institute for Health and Care Excellence (NICE) [4] does not recommend acupuncture. There have been inconsistent results in studies using acupuncture; researchers have proposed potential reasons for these inconsistencies, including the selection of acupoints, dosage, and the rationality of the sham acupuncture design [5]. Acupoint selection is a central component in clinical operation as well as a report standard [6]. In high-quality KOA studies, two acupoint selection methods were commonly used. One is composed of fixed acupoints (meaning that all participants in the acupuncture group were used) and optional acupoints [7, 8]. The other one only contained optional acupoints [9–11], that is, a certain number of acupoints was selected from them. Regardless of which method is adopted, acupoint selection varies greatly among studies. At present, the selection of acupoints mainly depends on traditional acupuncture theory and doctors’ clinical experience; this lack of a specific biological basis leads to different selections. According to research reports [12, 13], more than 40 acupoints can be used for KOA. However, little evidence is available about how many of these acupoints actually take effect, or which ones are more effective.

Some sensitizations can be found on the body surface of KOA patients, such as pain sensitization and thermal alteration. These sensitizations can guide both diagnosis and treatment [14, 15]. Acupoints also have the characteristics of sensitization. The biological characteristics of acupoints (such as skin temperature [16], tenderness threshold [17], and morphology [18]) may change in pathological conditions. Acupoints are specific points on the body surface, and sensitizations of a certain area should be similar to those of acupoints within this area, which provides a basis for acupuncture therapy. As a typical example, acupoint selection of dry needling can be guided by trigger points [19]. However, these links to skin temperature have not been explored clearly. Most acupoint selection methods guided by sensitization phenomena have large individual differences, with difficulties expanding. As a little-attention part of sensitization, skin temperature may be a more universal and objective factor to guide acupoint selection.

Infrared thermography (IRT) is a technology that collects focal plane temperature information within the field of view and displays surface temperature distribution in pseudocolor form [20]. IRT has high temperature sensitivity, which can reflect blood circulation and metabolism of tissue to be applied in medicine as a diagnostic test or outcome measurement [21]. Some abnormal skin temperature areas can be found in patients with KOA or other osteoarthritis [22, 23]. Recent evidence suggests that thermal alteration can reflect the progress of KOA and guide medical practice [15]. However, there is insufficient evidence to link these sensitized areas to acupoints. To further understand the characteristics of acupoints under pathological conditions, this cross-sectional case-control study will explore special manifestations of skin temperature of acupoints in patients with KOA by IRT. The results of the study will provide a theoretical basis for further optimizing the selection of acupoints.

## Objectives

### Primary objective

The primary objective of this study is to investigate the difference in the skin temperature of acupoints between KOA and healthy populations.

### Secondary objectives

The secondary objective of this study is to explore the influence of gender, painful area and pain level on the skin temperature of acupoints in KOA patients.

## Methods

### Study design

**Fig 1:**
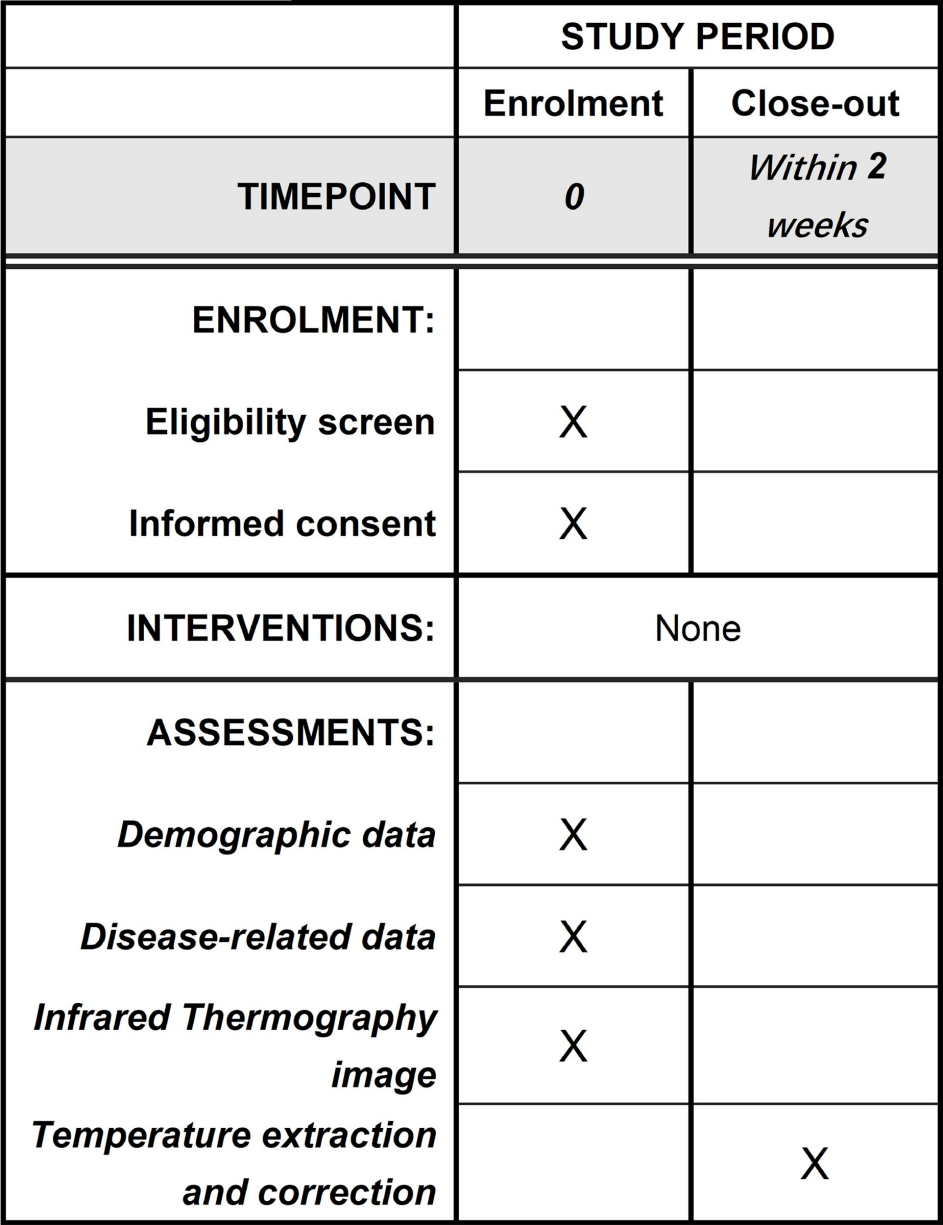
SPIRIT schedule.

This will be a cross-sectional case-control study conducted in Beijing, China. A total of 340 participants (170 KOA patients and 170 healthy individuals) will be recruited in Dongzhimen Hospital Affiliated to Beijing University of Chinese Medicine, Jiaodong Community Health Service Station, Xiaoguan East Street Community Health Service Station and Deluyuan Community Health Service Station. This study was approved by the ethics committee of Beijing University of Chinese Medicine (No. 2022BZYLL0304). The protocol (version number: V1.2, 2022/04/18) has been registered on the Chinese Clinical Trial Registry (ChiCTR) (registration number: ChiCTR2200058867). Written informed consent will be obtained from all participants before recruiting. Study results will be disseminated in peer-reviewed publications. This study will follow the Strengthening the Reporting of Observational Studies in Epidemiology (STROBE) [24] statement. Although this is an observational study, an adjusted SPIRIT schedule can be found in Fig 1 which clearly shows the overview of this study.

### Participants

Patients with KOA and age- and gender-matched healthy control individuals will be recruited in hospital and health service stations through doctors’ recommendations and print advertisements. Participants will be initially screened to determine if they meet the criteria. After signing the informed consent, eligible participants will be asked to complete a series of questionnaires and undergo an IRT examination.

### Inclusion criteria

The inclusion criteria for the KOA group are as follow: (1) meet the ACR clinical diagnostic criteria [25] of KOA; (2) aged 45–70 years old; (3) have unilateral/bilateral knee pain for more than 6 months; (4) have X-ray examination showed Kellgren-Lawrence grade [26] Ⅱ or higher within 6 months; (5) score 4 or high in numerical rating scale (NRS) during the past week for knee pain; (6) sign the informed consent.

The inclusion criteria for the healthy control group are as follow: (1) without knee pain in the past year; (2) aged 45–70 years old; (3) signed informed consent.

### Exclusion criteria

The exclusion criteria include (1) history of knee surgery; (2) knee pain caused by other diseases (such as severe effusion in the joint cavity, infection, malignant tumor, autoimmune disease, trauma, fracture, gout, lumbosacral disease, or rheumatic arthritis); (3) severe acute/chronic organic or psychoneurological diseases (such as epilepsy or severe anxiety/depression); (4) received drug therapy (such as nonsteroidal anti-inflammatory drugs or opioids) or nonpharmacologic therapy (such as acupuncture, moxibustion, infrared physiotherapy, or tuina) or any other treatment that may affect skin temperature distribution in the lower extremities during the past 2 weeks; (5) varicose veins, deep vein thrombosis, skin inflammation, allergic reactions or other factors that may affect the skin temperature distribution in the lower extremities; and (6) wearing excessive warm clothing or protective gear on the lower extremities for the past 24 hours.

### IRT measurement

#### Measuring locations

The skin temperature of 11 acupoints most commonly used for KOA will be measured [12]. The most commonly used acupoints include *dubi* (ST35), *neixiyan* (EX-LE5), *xiyangguan* (GB33), *yanglingquan* (GB34), *heding* (EX-LE2), *liangqiu* (ST34), *zusanli* (ST36), *xuanzhong* (GB39), *weizhong* (BL40), *yinlingquan* (SP9), and *xuehai* (SP10). The positioning of acupoints is shown in Table 1, which follows WHO Standard Acupuncture Locations.

**Table 1:** Positioning of acupoints.

Acupoint	Location
*Dubi* (ST35)	On the anterior aspect of the knee, in the depression lateral to the patellar ligament
*Neixiyan* (EX-LE5)	On the anterior aspect of the knee, in the depression medial to the patellar ligament
*Xiyangguan* (GB33)	On the lateral aspect of the knee, in the depression between the biceps femoris tendon and the iliotibial band, posterior and proximal to the lateral epicondyle of the femur
*Yanglingquan* (GB34)	On the fibular aspect of the leg, in the depression anterior and distal to the head of the fibula
*Heding* (EX-LE2)	On the anterior aspect of the thigh, in the depression superior to the base of the patella
*Liangqiu* (ST34)	On the anterolateral aspect of the thigh, between the vastus lateralis muscle and the lateral border of the rectus femoris tendon, 2 cun ^a^ superior to the base of the patella
*Zusanli* (ST36)	3 cun directly below ST35, and one finger-breadth lateral to the anterior border of the tibia
*Xuanzhong* (GB39)	On the fibular aspect of the leg, anterior to the fibula, 3 cun proximal to the prominence of the lateral malleolus
*Weizhong* (BL40)	On the posterior aspect of the knee, at the midpoint of the popliteal crease
*Yinlingquan* (SP9)	On the tibial aspect of the leg, in the depression between the inferior border of the medial condyle of the tibia and the medial border of the tibia
*Xuehai* (SP10)	On the anteromedial aspect of the thigh, on the bulge of the vastus medialis muscle, 2 cun superior to the medial end of the base of the patella

^a^ 1 cun (≈20 mm) is defined as the width of the interphalangeal joint of patient’s thumb.

#### IRT data acquisition specification

**Fig 2:**
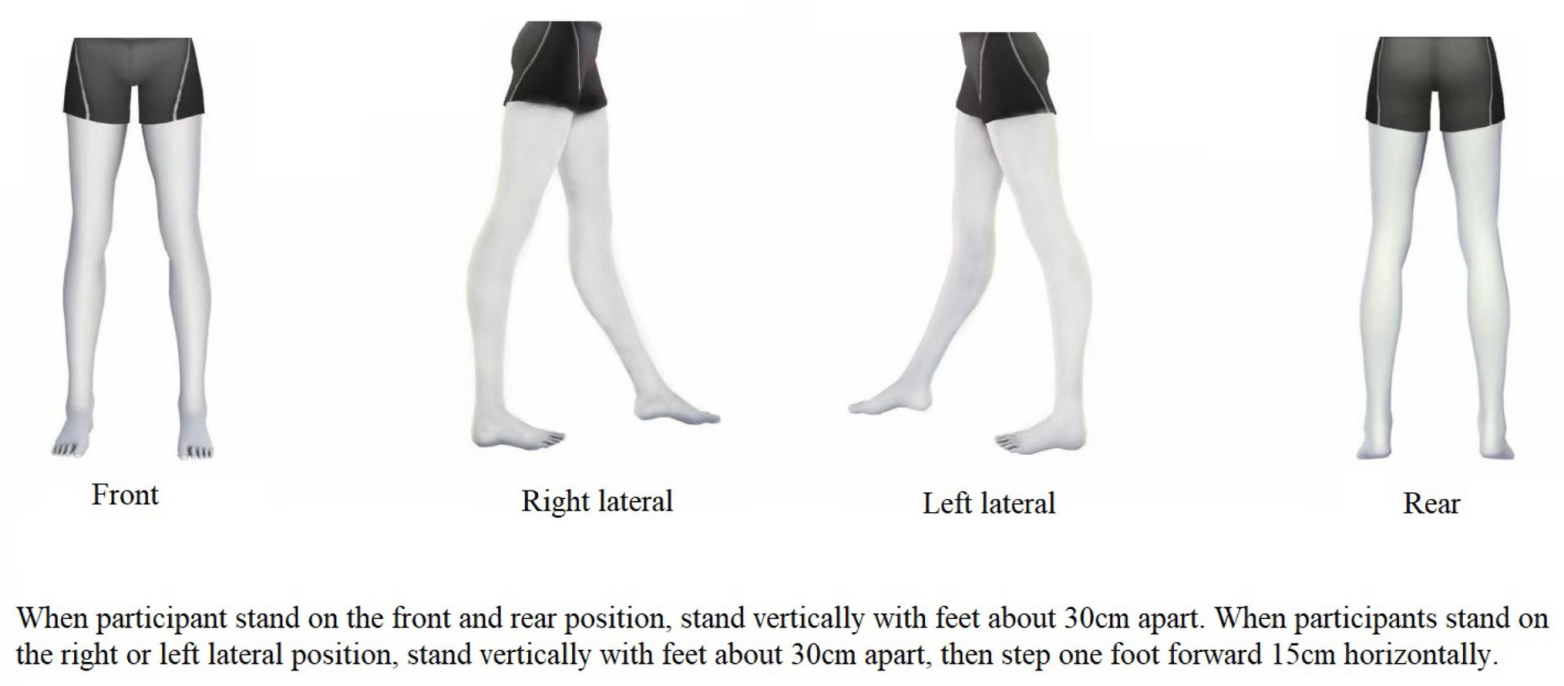
Standing posture of participants.

To ensure the standardization of data collection, data collection specifications referred to the Delphi study and consensus statement on the measurement of human skin temperature [27]. Similar specifications was reported in our previous study [28]. Specific specifications are as follows:

1. Personal information of all participants will be recorded in data sheets.
2. Participants will not be allowed to consume alcohol within 4 hours before the measurement.
3. Participants will not be allowed to conduct vigorous exercise, or to events that may severely interfere with skin temperature, such as electrotherapy, ultrasound, heat or cold exposure within two hours prior to measurement.
4. The ambient temperature will be controlled at 25±2°C, and the relative humidity of the air will be controlled at 40%-50%.
5. In the field angle of the infrared thermal image, there will be no interference heat source and obvious air flow.
6. The IRT detection equipment will be a TMI-M (Beijing Wholelife Medical Science Co., Ltd) portable medical infrared thermal imager. The thermal sensitivity is 0.05°C at 30°C, the spectral range is 7.5–13 μm, the image pixel size is 256×336, and the acquisition frequency is 25 Hz.
7. To make the equipment work in a steady state, the infrared thermal imager will be powered 0.5 hours before data collection.
8. The vertical distance between the participants and the camera will be 1.5 meters.
9. The centerline of the lens field angle will be perpendicular to the human body by a liftable gimbal.
10. The emissivity of the detector will be 0.98 (default value).
11. The image collection time for all participants will be 9:00–16:00.
12. All participants will stay in the same posture, as shown in Fig 2.
13. The IRT image data processing software was independently developed by the study team. This software has been verified to be reliable and maintains all original data characteristics. The data file contains temperature values of any spatial coordinates in all field angles. The data file is filled in a 16-bit integer data format and stored in *.dat.

### Extraction method for skin temperature of acupoints

**Fig 3:**
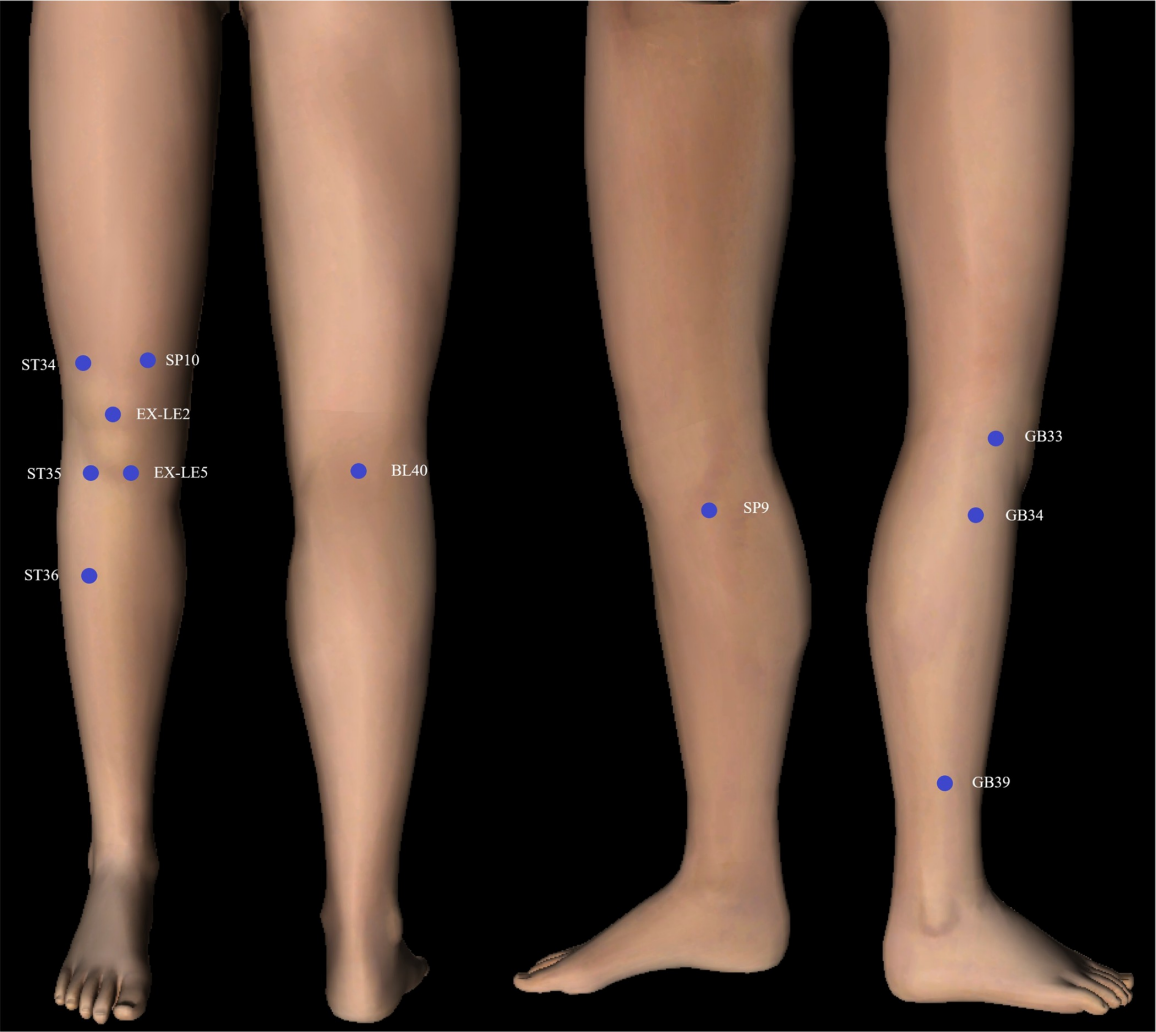
The location of the region of interest. The blue dots are centered on the acupoint positioning.

1. After obtaining the IRT image, a region of interest (ROI) will be drawn to extract the skin temperature of the acupoint. The IRT image contains both lower limbs. For the KOA group, the side with more severe symptoms will be used as the evaluation side. For the healthy control group, one side will be randomly selected for evaluation.
2. The size of the ROI is a circular area with a radius of 3 pixels centered on the acupoint positioning (fixed by software). The location of the ROI is shown in Fig 3.
3. The average value of the temperature will be extracted from the ROI; the value will be rounded up to 2 decimal places.
4. The ROI region will be drawn on the IRT images by 2 researchers independently. When the skin temperature of the acupoint is inconsistent, a third researcher will assist in discussing the ROI location.
5. IRT images can be presented in greyscale or pseudocolor. Since the pseudocolor image will weaken landmarks on the image, which will affect acupoint positioning, all ROI regions will be drawn on greyscale images.

### Privacy protection

Since participants will be required to take off their lower body clothes during IRT image collection, their privacy needs to be protected as follows:

1. The image collecting site will be separated from the outside environment by a movable screen.
2. When removing the lower clothes, participants will keep their underwear to maximize their privacy.

### Other measurements

#### Demographic data

Demographic data included gender, age, ethnicity, education, height, weight, BMI, etc. These data will be collected from all participants.

#### Disease-related data

Disease-related data included NRS [29], pain sites (front, back, outside or inside), duration of pain, pain descriptors (stabbing, bursting, sore, burning, etc.), pain activities (going upstairs, going downstairs, standing up, walking, etc.).

The primary outcomes included the relative skin temperature of 11 acupoints, and the secondary outcomes included the absolute skin temperature of 11 acupoints and other measurements.

### Sample size calculation

The relative skin temperatures of 11 acupoints are the primary outcomes of this study. The relative skin temperature of acupoints were preliminary extracted from the IRT images of 30 KOA patients and 30 healthy individuals at the clinic. The mean values of the KOA and healthy groups were 0.11°C and -0.23°C respectively, with the same standard deviation of 0.79°C in both groups. With 2-sided α = 0.0045 (adjusted for 11 primary outcomes using Bonferroni adjustment), 149 participants will be needed to provide 80% statistical power. Considering an attrition rate of 15%, a total of 340 participants (170 per group) are expected to be recruited.

### Data correction and statistical analysis

#### Correction of skin temperature

Human skin temperature is individual and affected by basal body temperature [30]. A previous study [31] showed that relative temperature, which can control bias from individual differences, is more valuable than absolute temperature under specific conditions. Relative temperature can eliminate the systematic error caused by factors such as body and ambient temperature, and truly express the temperature characteristics in the ROI. Using the quantification method of relative temperature, the temperature value in the ROI will be subtracted from the temperature value of other regions (reference areas) specified in the same image. In this study, at the same time that the absolute temperature will be reported, relative temperature will be analyzed as the main indicator. The specific correction formula for relative temperature is as follows:

$$T_{c}=T-\bar{T}$$

**Fig 4:**
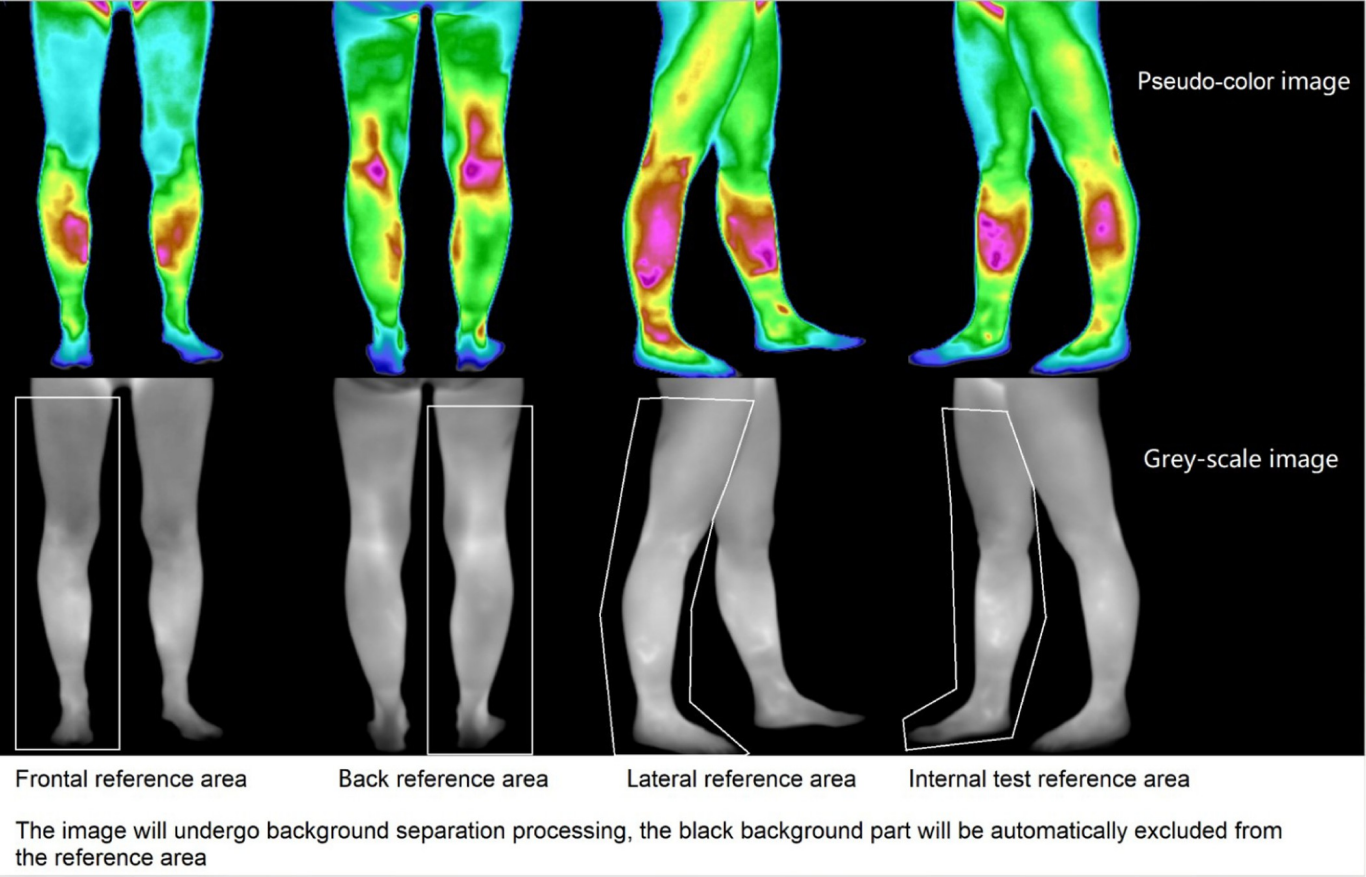
The location of reference areas.

*T_c_* is the measured value of the skin temperature of the acupoint after correction. *T* is the absolute skin temperature of the acupoint (the actual value measured). $\bar{T}$ is the control temperature, means the average skin temperature of the corresponding reference area. As shown in Fig 4, the reference area is the entire lower limb region on the IRT image which used to locate the acupoint.

#### Statistical analysis

For this study, the statistical analysts will be blinded. SAS version 9.3 (SAS Institute, Cary, NC, USA) or R software will be used for statistical analysis. Measurement data will be described as the mean ± standard deviation (M±SD) or median and interquartile ranges, and counting data will be described as frequency, constituent ratio or percentage. Independent samples t-tests or χ2 tests will be used to test the equilibrium of demographic data with a significance level of 0.05. The intraclass correlation coefficient (ICC) will be used to obtain the test-retest reliability of the temperature extracted by different researchers. A two-way random model with absolute agreement will be used for ICC calculation. An ICC value greater than 0.75 for each relative skin temperature of the acupoints evaluated will be considered to have good reliability. Comparison of relative skin temperature and absolute skin temperature between groups will be performed by Z test or χ2 test. For the relative skin temperature of acupoints, P < 0.0045 will be considered statistically significant. For other measurements, P < 0.05 will be considered statistically significant. Missing values and extreme values in demographic data will be confirmed and modified by phone calls before analysis. Other missing data will be inputted by the mean/mode completer.

Exploratory analysis will include the exploration of correlations and contributory factors within relative skin temperature at different acupoints. To simplify the factor structure of multiple relative skin temperatures of acupoints, when Kaiser-Meyer-Olkin (KMO) measurement value is > = 0.5 and Bartlett’s sphericity test value is <0.05, exploratory factor analysis will be adopted. After clustering and dimensionality reduction of temperature in two groups, the common factors will be extracted by principal component analysis. Then, the Kaiser normalized maximum variance method will be used to calculate the factor loading and factor score coefficient matrix to obtain the score of each common factors and acupoints’ relative skin temperature. A general linear regression model will be used to explore the influence some factors on skin temperature of acupoints. In the model, dependent variable will be the comprehensive factor scores; fixed factors will include age, gender, BMI, pain site and NRS, among which age and BMI will be transformed into qualitative data.

### Quality control

1. Participants will be enrolled in strict accordance with inclusion and exclusion criteria.
2. To have a unified standard for the operation of each link, experts discussed all aspects of the study and formulated relevant standard operating procedures.
3. Researchers will undergo unified training, including study objectives, inclusion and exclusion criteria, IRT hardware and software operation, case report form, etc. Researchers with the same division of labor need to pass the consistency test before the study begins, and the test will be saved by video data.
4. Uniformly printed case report forms will be used in strict accordance with the study design and filled in seriously and objectively. All kinds of problems in the process of data collection will be recorded truthfully.
5. The integrality of records and data collection will be examined every 20 participants enrolled or after 1 month.

## Discussion

Although it is widely used, acupuncture carries a high burden for some elderly groups in many countries [32, 33]. The theoretical basis and clinical effect of acupuncture in treating KOA need to be improved. As the main symptom of KOA, pain intensity has a strong correlation with skin temperature [34]. KOA involves a variety of inflammatory factors, under which the dynamic alteration between the repair and destruction of joint tissues becomes lopsided [35]. These changes in local tissue resulting from the inflammatory response can appear in skin temperature. Overgrowth of angiogenesis is the underlying cause of pain [36], and this change in hemodynamics can also be detected by IRT. At present, the relationship between these abnormal areas of skin temperature and acupoints is not clear. Dong Zhang’s [37] study found that in acupuncture treatment of peripheral facial paralysis, compared with conventional acupoint selection, selecting acupoints with abnormal skin temperature provides a better effect. Therefore, selecting acupoints by thermography might be a valuable method. We suppose that skin temperature at some of the included acupoints in the lower extremity region of KOA patients will be different from that of healthy individuals in the results. This will help clinical practice to select more valuable points from a large number of available acupoints.

This study is only the beginning of a series of studies aimed at improving acupoint selection and providing a biological basis, which is conducive to the effect of acupuncture. The results of this preliminary study will provide evidence on which acupoints have abnormal skin temperature in KOA patients. According to some other sensitizations of acupoints that can guide acupoint selection [38], we reasonably infer that the characteristics of skin temperature have instructive value as well. Following this study, a randomized controlled trial comparing conventional acupoint selection with skin temperature-guided acupoint selection for KOA will be designed to verify our hypothesis.

To our knowledge, this is the first study to investigate the skin temperature of acupoints in KOA patients. IRT technology has the characteristics of noninvasive, nonradiation, noncontact and high repeatability. It is currently one of the best methods to measure the skin temperature of acupoints. For inflammatory diseases such as KOA, IRT can simply and clearly reflect the condition and guide medical treatment [15]. This will be a multicenter study in which patients will be recruited from primary and tertiary hospitals simultaneously. Patients included in this study will be a typical KOA population with any KL grade other than grade I, which is undiagnosable. Patients who underwent knee surgery or report NRS scores less than 4 will be excluded because we cannot ensure the stability of the lower extremity temperature in this population. Acupoints assessed will be the most common ones in clinical practice. Moreover, two temperature correction methods, an adequate sample size and a strictly corrected P value increase the credibility of this study.

This study still has a few limitations. First, the location of the ROI drawn by different researchers may be slightly different since the positioning of acupoints are rough positions rather than precise points. However, ROIs will be drawn repeatedly by different researchers to ensure the consistency of acupoint location. Second, this study only focused on skin temperature at acupoints, which is of limited reference value to therapies other than acupuncture.

## Supporting information

S1 File SPIRIT checklist. (DOCX)

S2 File Original protocol approved by the ethics committee. (DOCX)

## Notes Abbreviations

KOA: knee osteoarthritis
IRT: infrared thermography
OARIS: Osteoarthritis Research Society International
ACR: American College of Rheumatology
NICE: National Institute for Health and Care Excellence
ChiCTR: Chinese Clinical Trial Registry
STROBE: Strengthening the Reporting of Observational Studies in Epidemiology
NRS: numerical rating scale
ROI: region of interest
ICC: intraclass correlation coefficient
