# Supplementary material for: Thermographic evaluation of acupoints in lower limb region of individuals with osteoarthritis: A cross-sectional case-control study protocol
Source: PLoS One. 2023 Apr 14;18(4):e0284381. doi: 10.1371/journal.pone.0284381 (PMC10104310; doi:10.1371/journal.pone.0284381)
Supplement: S2 File — (DOCX) [file pone.0284381.s002.docx]

**Clinical Study Protocol (The translated version)**

**Investigation of acupoints’ temperature in patients with knee osteoarthritis: a cross-sectional matched case-control study**

**The study contents**

Objective: To explore the temperature distribution characteristics of commonly used acupoints in patients with knee osteoarthritis

Participants: Patients with knee osteoarthritis and healthy people

Intervention: None

Data of the study includes demographic data, skin temperature of acupoints, disease-related data of patients with knee osteoarthritis; demographic data and skin temperature of acupoints of healthy people.

Study design: Cross-sectional study

Study period: January 2022 to December 2022

**Background**

Knee Osteoarthritis (KOA) is one of the most common musculoskeletal diseases, and it is also an important cause of chronic pain and lower limb disability in the elderly. The situation will further worsen with the extension of human average life span and the epidemic of obesity [1]. The common symptoms of KOA are pain, swelling, joint stiffness or deformity, and dysfunction, etc. The main pathological features are cartilage degeneration and destruction of the knee joint, subchondral bone sclerosis, synovial lesions, and bone hyperplasia at the joint edge [2].KOA seriously reduces the mobility of patients, affects the emotional and mental state of patients, and severe patients may lose their ability to work, which brings a huge economic burden to patients and society [3].The population with KOA is huge, and the lifetime risk of KOA is about 40% in men and 47% in women [4].The incidence of symptomatic KOA in China can reach 8.1%[5-6].In Europe, the incidence of symptomatic KOA patients ranges from 1.6% to 14.9% according to different ages [7].

Clinically, symptomatic treatment is mostly used for mild to moderate KOA, and surgical treatment is used for severe KOA [8-10]. The short-term efficacy of drug therapy for KOA is good. However, KOA often recurs with the extension of drug withdrawal time, and some patients have different degrees of adverse reactions. Therefore, the guidelines put non-drug therapy in the first place and emphasize the importance of non-drug therapy. [8-10]. Acupuncture is a widely recognized non-drug therapy for the treatment of KOA at home and abroad. Meta-analysis and large-scale randomized controlled trials have shown that acupuncture can effectively improve the pain symptoms in KOA patients [11-14]. There are many factors affecting the efficacy of acupuncture, the selection of acupoints is one of the most important factors affecting the clinical efficacy. According to the review of acupoint selection rules and relevant research reports of expert consensus, there are more than 40 acupoints that can be used for the treatment of KOA, of which 11 are the most commonly used [15-17]. However, the clinical evidence for the difference these acupoints in the treatment of KOA is not clear. At present, acupoint selection mainly based on medical literature and clinical experience, with individual differences between patients and doctors. The correlation between these acupoint related characteristics and the disease state of KOA and the difference of acupoint characteristics between KOA population and healthy people have not been reported. This correlation and difference investigation may provide basis and clues for clinical acupoint selection.

Infrared Thermography (IRT) technology is an imaging device that can collect the temperature information of the focal plane in the field of view angle, and display the temperature distribution information of the object surface in pseudo-color form [18].IRT technology has high temperature sensitivity, which can locate the abnormal areas of subcutaneous blood circulation and tissue metabolism according to the temperature difference of human skin, and reflect the local tissue metabolism [19].I In view of this, we set out to explore the changes in skin temperature at acupoints commonly used for the treatment of KOA; the relationship between acupoint temperature and disease state parameters such as pain location, pain feelings, BMI, and disease duration; the differences between groups at the same acupoint .Therefore, through a cross-sectional study, this study collected the IRT data of the lower limbs, then extracted the data of commonly used acupoint temperature. Combined with the disease related situation of the participants, the correlation between the distribution of acupoint temperature and the disease state parameters in the KOA group will be investigated. At the same time, a group of healthy participants matched by gender and age will be include. The difference in temperature distribution of the same acupoint between the two groups will be observed, then the temperature distribution pattern and law of lower limb acupoint in KOA group will be found. This is conducive to finding objective clues for optimizing acupoint selection at the macro level, and also lays a foundation for the next step of optimizing acupoint selection intervention research.

**Study protocol**

**(1) Participants**

Dongzhimen Hospital Affiliated to Beijing University of Chinese Medicine, Jiaodong Community Health Service Station, Xiaoguan East Street Community Health Service Station and Deluyuan Community Health Service Station participate in this study. Participants join this study through wechat, print advertisements, and recruitment notices posted in the outpatient clinic.

1. Inclusion criteria

KOA group:

① meets the clinical criteria of knee osteoarthritis of the American College of Rheumatology [20];

② Age 45-75 years old;

(3) unilateral/bilateral knee pain more than 6 months;

④ X-ray examination within six months showed Kellgren-Lawrence grade II or above;

Grade 0: normal;

Grade ⅰ: suspected narrowing of joint space and possible osteophytes;

Grade ⅱ: obvious osteophytes and suspected narrowing of joint space;

Grade ⅲ: moderate osteophytes, narrowing of joint space, and sclerotic changes;

Grade ⅳ: a large number of osteophytes, obvious narrowing of joint space, severe sclerosis and obvious deformity.

⑤ Average Numeric Rating Scale (NRS) scores higher than 4 points during the past 7 days;

⑥ Willing to sign informed consent.

Health groups:

① No knee pain in the past year;

② Age 45-75 years old;

③ Willing to sign informed consent.

2. Exclusion criteria

① Patients with a history of knee surgery;

(2) knee pain caused by other diseases (such as joint free bodies, serious joint cavity effusion, infection, malignant tumors, autoimmune diseases, trauma, fracture, gout, lumbosacral diseases, rheumatoid arthritis, etc.);

③ Participants with severe acute or chronic organic or psychoneurotic diseases (such as epilepsy, severe anxiety/depression, etc.);

④ Participants who received acupuncture, pain relief (acetaminophen, tramadol, non-steroidal anti-inflammatory drugs, etc.) or nutritional cartilage (glucosamine, etc.) in the past 2 weeks;

⑤Participants with lower extremity varicose veins, deep vein thrombosis, skin inflammation, allergic reaction or other affecting skin temperature distribution;

⑥ Participants who had received moxibustion or infrared physiotherapy in the lower limbs within the past 3 days;

⑦ Participants who wear local warm clothing or protective equipment of lower limbs such as knee pads within one day;

⑧ Participants with obvious redness, swelling and heat pain in the lower limb knee joint;

Those fulfilling one or more of the above requirements will be excluded.

**(2) Investigation contents**

**1. The lower limb acupoint temperature of the participants**

(1) Measuring acupoints

The 11 most commonly used acupoint temperature characteristics and 15 available acupoint temperature characteristics of KOA patients will be extracted, and the specific acupoint list is as follows:

The most commonly used acupoints are Dubi (ST35), Neixiyan (Ex-Le5), Xiyangguan (GB33), Yanglingquan (GB34), Heding (Ex-LE2), Liangqiu (ST34), Zusanli (ST36), Xuanzhong (GB39), Weizhong (BL40), Yinlingquan (SP9), Xuehai (SP10).

Available acupoints: Ququan (LR8), Fenglong (ST40), Futu (ST32), Fengshi (GB31), Waiqiu (GB36), Weiyang (BL39), Chengshan (BL57), Kunlun (BL60), Taixi (KI3), Yingu (KI10), Gongsun (SP4), Sanyinjiao (SP6), Taichong (LR3), Xiguan (LR7) Sobbing at the foot (GB41). This part of the acupoints will be used as the data for secondary analysis.

(2) Measurement methods

A. When drawing the region of interest (ROI) on IRT images to extract the temperature of the acupoint, two researchers independently completed it first. When the temperature of the acupoint are inconsistent, the location of the ROI will be discussed together.

B. Size of ROI: a circular area with a radius of 5pixel units (fixed by the software);

C. The temperature of the acupoint will be rounded to retain two decimal places, and the maximum, average and minimum values will be extracted.

D, the location of ROI will be selected according to Figure 1;

E The average point temperature of ROI will be extracted as a quantitative parameter.


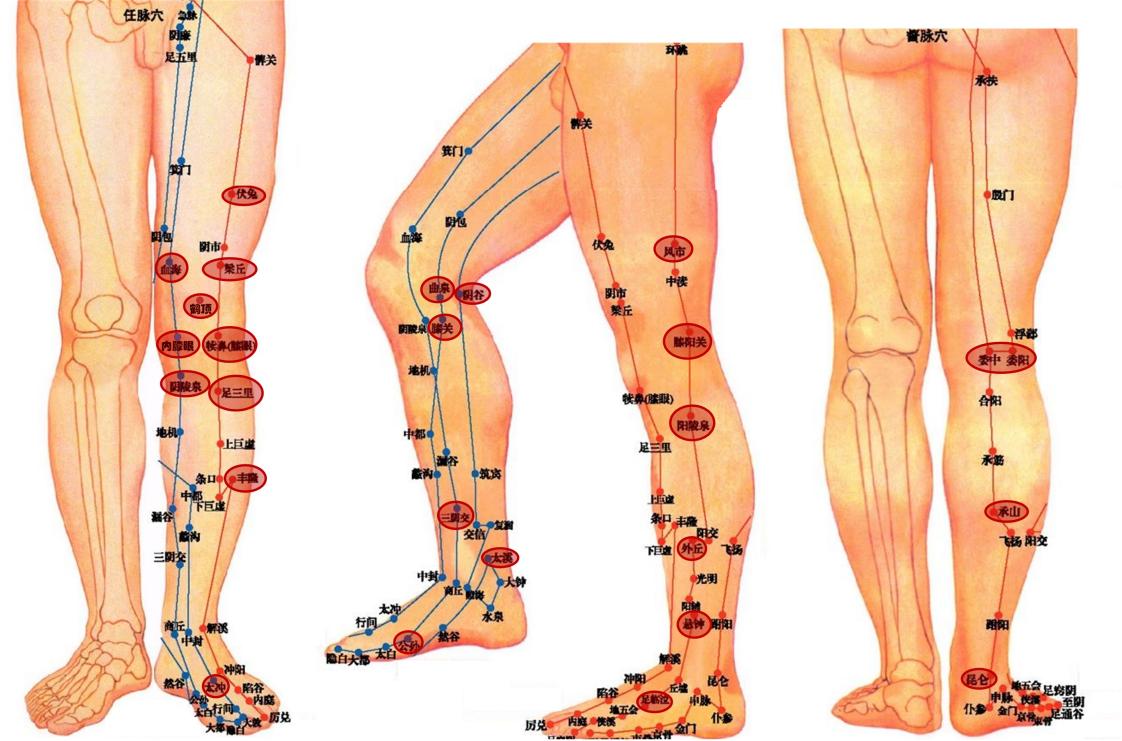


Figure 1 Reference position of acupoints

**2. General information of participants**

(1) Demographic data

Including: gender, age, nationality, education level, height, weight, BMI.

(2) Information about disease status of KOA patients

It includes: pain rating scale (NRS) [21], pain location (anterior and posterior, internal and external), pain duration, pain nature (swelling pain, tingling pain, cold pain, burning pain, aching pain, etc.), responsible movements (upstairs pain, downstairs pain, continuous pain, etc.).

(3) Medical records

The patient's past medical history and medication records will be included.

**3. Environmental information**

Including: ambient temperature, humidity, etc.

**(3) Sample size estimation**

According to the image data of 30 KOA patients and healthy people collected earlier, the relative temperature of Dubi point was obtained. The mean of the KOA group was 0.11, and the standard deviation was about 0.79, and the mean of the healthy group was -0.23, and the standard deviation was about 0.79. The α was set to 0.0045 (corrected by 11 measurement points), 1-β was 0.2. The sample size ratio of the two groups will be 1:1, and the sample size of each group could be 149. Considering the non-response rate of 15%, a total of 170 KOA patients and 170 healthy people need to be included.

**(4) IRT data collection**

In order to ensure the standardization of data collection, this study referred to the Delphi study and consensus statement on human skin temperature measurement proposed in literature [22], and the data collection specification is as follows:

1. Personal information of all participants will be recorded in data sheets.

2. Participants will not be allowed to intake alcohol within 4 hours before the measurement.

3. Participants will not be allowed to conduct vigorous exercise, or to events that may severely interfered with skin temperature, such as electrotherapy, ultrasound, heat or cold exposure within two hours prior to measurement.

4. Ambient temperature will be controlled at 25±2℃, and relative humidity of the air will be controlled at 40%-50%.

5. In the field angle of infrared thermal image, there will be no interference heat source and obvious air flow.

6. IRT detection equipment will be TMI-M (Beijing Wholelife Medical Science Co., Ltd) portable medical infrared thermal imager. The thermal sensitivity is 0.05℃ at 30℃, the spectral range is 7.5-13μm, the image pixel size is 256×336, and the acquisition frequency is 25Hz.

7. To make the equipment work in steady state, the infrared thermal imager will be powered on 0.5 hours before data collection.

8. The vertical distance between the participants and the camera will be 1.5 meters.

9. The center line of the lens field angle will be perpendicular to the human body by liftable gimbal.

10. Emissivity of the detector will be 0.98 (default value).

11. Image collection time for all participants will be 9:00-16:00.

12. All participants kept the same standing posture, as shown in Figure 2.

13. The infrared thermal image data processing software is independently developed by our team, and the data saving format is *.dat file, which contains the temperature values of any spatial coordinates in all field angles and is filled in 16-bit integer data format.

Front Right Left Back

FIG. 2 Unified standing posture of participants

**(5) Privacy protection**

In view of the fact that participants need to remove their lower body clothing during infrared image acquisition, it is necessary to protect the privacy of participants, and the specific protective measures are as follows:

(1) The infrared image shooting site will be isolated from the external environment by a movable screen.

(2) If the hospital has a cabin type infrared thermal imaging device, the portable camera selected in this project will be placed in the cabin to complete image acquisition;

(3) When the participants take off their lower body clothes, they can keep their underwear to protect the participants' privacy to the greatest extent.

**(6) Data entry and statistical analysis**

1. SAS 9.3 or R software will be used for statistical analysis. Measurement data will be expressed as mean ± standard deviation (M±SD) or median and interquartile range, and count data will be expressed as frequency, constituent ratio and percentage.

2. Independent sample t test or χ2 test will be used to test the balance of demographic data. The test level will be 0.05, that is, P < 0.05 will be considered to indicate statistical significance.

3. Conduct internal and external reliability tests on the acupoint temperature data extracted by different researchers. The reliability test will be obtained by ICC.

4. Describe the acupoint temperature of patients with different pain sites, different course of disease and different nature of pain.

5. Single factor linear regression analysis will be performed on the pain rating scale, pain location, pain duration, pain nature, responsible action and different acupoint temperatures to screen the influencing factors of acupoint temperature.

6. According to the influencing factors after screening, multiple linear regression models will be constructed for different acupoints to test the correlation between the temperature of different acupoints and the pain score scale, pain location, pain duration, pain nature and responsible action.

7. The linear mixed effect model will be used to test the difference of acupoint temperature between the two groups. The random effect will be the individual patient, the fixed effect will be the acupoint temperature, and the pain site, pain nature, pain duration, responsible action and NRS score will be used as covariates.

8. Subgroup analysis: Subgroup analysis will be performed according to gender and unilateral or bilateral disease.

**(7) Quality control**

①The inclusion of participants will be strictly according to the diagnosis, inclusion and exclusion criteria.

② After expert discussion, relevant standard operating procedures will be formulated for each link of the test, so that the operation of each link has a unified standard, and there is a basis to follow when opinions are inconsistent.

③ Researchers must undergo unified training. The content includes being familiar with the objectives and requirements of this study, mastering the relevant diagnosis and treatment standards, the operation process of infrared thermal imaging software system, the use of evaluation forms, etc. Researchers with the same division of labor in the research center need to pass the consistency test to carry out the trial, and the video data of consistency test are required to be stored.

④ The case questionnaire will be printed uniformly, the operation will be carried out in strict accordance with the design scheme of the participant, the questionnaire will be filled out seriously and objectively, and the various problems occurred in the process of collecting poetry will be recorded faithfully.

⑤ Secondary confirmation and modification of missing values, extreme values, special symbols, etc. by telephone.

⑥ Strengthen compliance control. Patients will be fully informed of the purpose and significance of the study, and signed an informed consent form. Related examinations will be free of charge, and the cost will be paid by the project fund.

⑦ After every 20 participants will be included or after 1 month, the monitor randomly detected the record and data collection of the study record book. In case of problems, timely report to the superior, find problems, solve problems and strictly implement.

**reference**

[1]Emery C A , Whittaker J L , Mahmoudian A , et al. Establishing outcome measures in early knee osteoarthritis[J]. Nature Reviews Rheumatology, 2019, 15(6).

[2]Michael JW, Schluter-Brust KU, Eysel P. The Epidemiology, etiology, Diagnosis, and treatment of osteoarthritis of the knee. Dtsch Arztebl Int. 2010 Mar;107 (9) : 152-62. The doi: 10.3238 / arztebl. 2010.0152.

[3]Jones I A , Togashi R , Wilson M L , et al. Intra-articular treatment options for knee osteoarthritis[J]. Nature Reviews Rheumatology, 2018.

[4]Johnson V L, Hunter D J. The epidemiology of osteoarthritis[J]. Best practice & research Clinical rheumatology, 2014, 28 (1) : 5-15.

[5] Chinese Orthopedic Society Joint Surgery Group. Guidelines for the diagnosis and treatment of osteoarthritis (2018 edition) [J]. Chin J Orthopaedics,2018,38(12): 705-715.

[6] Tang X, Wang S, Zhan S, et al. The Prevalence of Symptomatic Knee Osteoarthritis in China: Results From the China Health and Retirement Longitudinal Study. Arthritis Rheumatol. 2016;68:648-53.

[7]Guillemin F, Rat AC, Mazieres B, et al. Prevalence of symptomatic hip and knee osteoarthritis: a two-phase population-based survey. Osteoarthr Cartil. 2011;The nobleman 14-22.

[8]Hochberg MC, Altman RD, April KT, et al. American College of Rheumatology 2012 recommendations for the use of nonpharmacologic and pharmacologic therapies In osteoarthritis of the hand, hip, and knee[J].Arthritis Care Res (Hoboken), 2012,64(4):465-74.

[9] Zhang W, Nuki G, Moskowitz RW, et al. OARSI recommendations for the management of hip and knee osteoarthritis: part III: Changes in evidence following systematic cumulative update of research published through January 2009[J]. Osteoarthritis Cartilage, 2010, 17 (4) : 476-99.

[10]Osteoarthritis: Care and Management in Adults. London:National Institute for Health and Care Excellence(UK),2014.

[11]Lin X, Huang K, Zhu G,et al. The Effects of Acupuncture on Chronic Knee Pain Due to Osteoarthritis: A Meta-Analysis[J]. J Bone Joint Surg Am. 2016 Sep 21;98 (18) : 1578-85.

[12]Corbett MS, Rice SJ, Madurasinghe V, et al. Acupuncture and other physical treatments for the relief of pain due to osteoarthritis of the knee: network meta-analysis[J]. Osteoarthritis Cartilage. 2013;21 (9) : 1290-8.

[13]Scharf H P, Mansmann U, Streitberger K, et al. Acupuncture and knee osteoarthritis: a three-armed randomized trial[J]. Annals of internal medicine, 2006, 145(1): 12-20.

[14]Tu JF, Yang JW, Shi GX, et al. Efficacy of Intensive Acupuncture Versus Sham Acupuncture in Knee Osteoarthritis: A Randomized Controlled Trial. Arthritis Rheumatol. 2021 Mar;73 (3) : 448-458. The doi: 10.1002 / art. 41584.

[15]Sun N, Wang LQ, Shao JK, Zhang N, Zhou P, Fang SN, Chen W, Yang JW, Liu CZ. An expert consensus to standardize acupuncture treatment for knee osteoarthritis. Acupunct Med. 2020 Oct;38 (5) : 327-334. The doi: 10.1177/0964528419900789.

[16] Zou Yuan, Zhu Xiangyu, Chen Yafei, Wei Bowen, Liu Zidan, Li Qiao-qiao, Chen Yuan-lin, Huang Yi-ran, Li Wenxun. Exploration of acupoint selection rules of acupuncture and moxibustion for knee osteoarthritis based on traditional Chinese Medicine inheritance assistance platform [J]. World journal of integrated Chinese and western medicine,2021,16(08):1406-1410+1420.

[17]Gong Z, Liu R, Yu W, Wong TK, Guo Y, Sun Y. Acutherapy for Knee Osteoarthritis Relief in the Elderly: A Systematic Review and Meta-Analysis. Evid Based Complement Alternat Med. 2019 Feb 17;2019:1868107. Doi: 10.1155/2019/1868107.

[18]Baohong Mi, Jialin Song, Wenxue Hong, et al. Evaluation method of infrared thermography on children with idiopathic thrombocytopenic purpura: [J]. Infrared Physics & Technology,2019, 102:103027.

[19]Ring E F J, Ammer K. Infrared thermal imaging in medicine[J]. Physiological measurement, 2012, 33(3): R33.

[20]Hochberg M C,Altman R D,Brandt K D et al. Guidelines for the medical management of osteoarthritis. Part I. Osteoarthritis of the hip. American College of Rheumatology.[J] .Arthritis Rheum, 1995, 38: 1535-40.

[21]Hawker Gillian A,Mian Samra,Kendzerska Tetyana et al. Measures of adult pain: Visual Analog Scale for Pain (VAS Pain), Numeric Rating Scale for Pain (NRS Pain), McGill Pain Questionnaire (MPQ), Short-Form McGill Pain Questionnaire (SF-MPQ), Chronic Pain Grade Scale (CPGS), Short Form-36 Bodily Pain Scale (SF-36 BPS), and Measure of Intermittent and Constant Osteoarthritis Pain (ICOAP).[J] .Arthritis Care Res (Hoboken), 2011, null: S240-52.

[22]Moreira, D. G., Costello, J. T., Brito, C. J., et al. Thermographic imaging in sports and exercise medicine: A Delphi study and consensus statement on the measurement of human skin temperature[J]. Journal of Thermal Biology, 2017, 69:155-162.
